# Supplementary material for: MetaRibo-Seq measures translation in microbiomes
Source: Nat Commun. 2020 Jun 29;11:3268. doi: 10.1038/s41467-020-17081-z (PMC7324362; doi:10.1038/s41467-020-17081-z)
Supplement: Supplementary file 10 — Supplementary Data 7 [file 41467_2020_17081_MOESM10_ESM.zip › File2/Confidence_High_Taxonomy/201552_out.krona.html]

Javascript must be enabled to view this page.

members
magnitude
magnitudeUnassigned
count
unassigned
taxon
rank

201552\_out

5


SRS023914\_contig\_number\_28057SRS051031\_contig\_number\_36169
superkingdom
2
2759
5

4751
kingdom
3

species
1427494

SRS1041145\_contig\_number\_9323
1

1913637
phylum
2

1137986
subphylum
2

class
2212732
2

214503
order
2

2
family
4854

4855
genus
2

310910
species

SRS013687\_contig\_number\_18511SRS053356\_contig\_number\_contig-100\_1985.246856
2
